# Deep Eutectic Solvents and Nonconventional Technologies for Blueberry-Peel Extraction: Kinetics, Anthocyanin Stability, and Antiproliferative Activity

**DOI:** 10.3390/antiox9111069

**Published:** 2020-10-31

**Authors:** Giorgio Grillo, Veronika Gunjević, Kristina Radošević, Ivana Radojčić Redovniković, Giancarlo Cravotto

**Affiliations:** 1 Dipartimento di Scienza e Tecnologia del Farmaco, University of Turin, 10235 Turin, Italy; giorgio.grillo@unito.it (G.G.); veronika.gunjevic@unito.it (V.G.); 2 Department of Biochemical Engineering, Laboratory for Cell Culture Technology and Biotransformations, Faculty of Food Technology and Biotechnology, University of Zagreb, 10000 Zagreb, Croatia; irredovnikovic@pbf.hr

**Keywords:** blueberry peels, anthocyanins, extraction kinetics, NADES, ultrasound, microwave, antiproliferative activity

## Abstract

Interest in bioactive phytochemicals and sustainable processes is the driving force behind this study on two novel green extraction methods for the recovery of anthocyanins from the residues of blueberry processing. Five natural deep eutectic solvents (NADES) have been tested for anthocyanin extraction. Acidified hydroalcoholic solutions were used as benchmarks and the shelf life of eutectic systems was monitored. The most promising NADES was tested in microwave (MAE)- and ultrasound-assisted extractions (UAEs), and Peleg’s kinetic model was used. Both the enabling technologies provided performance that was superior to that of conventional extraction. MAE and UAE yielded up to 25.83 and 21.18 mg/g_matrix_ of total anthocyanin content, respectively, after 15 and 30 min. Moreover, a preliminary test for extract concentration and NADES recycling was performed using resin adsorption. Finally, the antiproliferative activity of the extracts was determined by a CellTiter 96^®^ AQ_ueous_ One Solution Cell Proliferation Assay, the so-called MTS assay, on human tumour HeLa cells and human skin HaCaT cells. Nonconventional extracts exhibited strong antiproliferative activity that was much greater than that of their conventionally extracted analogues. Flow cytometry was used to evaluate cell-death type, and apoptosis was found to be the primary cause of tumour cell death. The presented study demonstrates that the implementation of enabling extraction technologies and green solvents can produce an antiproliferative agent from a food industry byproduct.

## 1. Introduction

Waste minimisation via reuse and recycling is one of the main principles of the circular economy [1,2]. Reuse and recycling are preferable alternatives to currently prevailing waste management methods and are capable of furnishing increases in economic profit together with a reduction in environmental impact. The food industry generates large amounts of waste and byproducts that are usually composted or simply disposed of in open fields, causing environmental pollution due to high chemical- and biological-oxygen demand [3,4].

Blueberry peels (BP) are one of the main byproducts of blueberry fruit processing. Blueberries have increased in popularity in recent years thanks to their health benefits, nutritional value, and sensory properties. In particular, they possess some of the highest antioxidant activities of all fruits, which is mainly due to their exceptional concentration of anthocyanins [5]. Furthermore, it is worth noting that the peels have the highest content of these metabolites, compared to the remaining berry components [6].

Anthocyanins are water-soluble plant pigments that belong to the widespread flavonoid group of polyphenols [7]. The beneficial health effects of dietary anthocyanins have been demonstrated in many in vivo and in vitro studies, and they have been included in epidemiological and clinical research in human volunteers [7,8]. Their strong antioxidant activity means that anthocyanins have a positive effect on the prevention of cardiovascular disease, while they also show antiproliferative, anti-inflammatory, and neuroprotective activity [8,9]. Cancer is the second leading cause of mortality, as it led to 9.6 million deaths globally in 2018 [10]. As chemotherapy and many anticancer drugs that are currently available have potential life-threatening side effects, it is necessary to find new, less dangerous agents [11]. As a result, chemoprotection by naturally occurring compounds has gained increasing attention [12], and anthocyanins can be considered promising chemoprotective agents, especially for tumorigenesis that occurs at directly accessible targets, such as the gastrointestinal tract and skin [13].

Due to their impressive health-promoting properties, worldwide anthocyanin assets are constantly growing. The global market size was valued at USD 318 million in 2019 and is expected to increase at a compound annual rate of 4.6% from 2019 to 2024 [14]. In addition, anthocyanins are used as natural pH-dependent colourants in the food industry. These pigments appear to be red or pink in acidic solutions and blue to purple in alkaline conditions [15]. Besides colour structural modification, acidity can greatly affect the overall stability of anthocyanins, leading to degradation at pH > 7 [16]. Generally, these flavonoids are highly unstable and very prone to degradation, as they are also sensitive to storage temperature, oxygen, light exposure, and even the presence of enzymes [17]. This behaviour worsens in isolated and concentrated compounds [18]. According to the literature, anthocyanin stability is often enhanced by the addition of natural compounds, such as other polyphenols and organic acids, during or after processing [17,19,20]. Furthermore, many researchers have investigated how cold storage can influence the degradation behaviour and antioxidant capacity of blueberries [21,22], but there is huge variability in the results and trends. Food processing and storage can therefore dramatically alter the final products, restricting their use in dietary applications.

Highly efficient solid–liquid extraction facilitates the convenient recovery of high-value secondary metabolites from food byproducts [23,24]. Firstly, the pretreatment, such as grinding and drying, of plant material must be considered. The core process then requires post-treatment such as filtration, drying, and purification. Extraction is the crucial unit operation and, when this is not optimised, can lead to high energy consumption, as well as low extraction yield and selectivity. Furthermore, environmental pollution and safety issues can be generated if volatile organic solvents are used [24]. Therefore, in past decades, efforts have been made to develop sustainable, safe, and efficient extraction methods, while economic, social, and environmental requirements must also be considered [25]. Numerous protocols for anthocyanins have been presented to date, and these include Soxhlet extraction, ultrasound-assisted extraction (UAE), microwave-assisted extraction (MAE), supercritical fluid extraction, pulsed electric field extraction, etc. [26].

UAE and MAE belong to the class of enabling technologies for process intensification. They provide time and energy savings, increased yields, and safe and high-quality extracts [24]. Ultrasound (US) is a nonthermal technology, which principally exploits cavitation phenomena, and is widely used in the food industry [23]. Cavitation effects enhance mass transfer, causing cell rupture and the release of cytoplasmic material into the solvent [27,28]. Conversely, MAE provides decreases in extraction time and high extraction yields thanks to the synergic effect of mass and heat transfer phenomena [29]. Unlike conventional conduction heating, microwaves (MW) heat matter in a selective and targeted manner, giving homogeneous temperature profiles, and are an energy-effective approach [30,31].

The same goal is currently being pursued by the scientific and industrial community through the development of new, environmentally friendly bio-based solvents that can improve extraction processes efficiency and selectivity [24]. Natural deep eutectic solvents (NADES) are under the spotlight as they are potential alternatives for conventional organic solvents [32]. NADES are a new generation of ionic liquids formed by mixing two or more naturally occurring components, such as cholinium chloride, used as a hydrogen bond acceptor, and sugar, alcohol, amino acids, vitamins, organic acids, and organic bases as hydrogen bond donors [32,33]. Since their constituents are naturally occurring, they are inherently nontoxic and environmentally benign [34]. These features imply that the incorporation of NADES into food formulations without additional purification and separation steps is feasible [35]. Their physicochemical properties can be tailored thanks to several possible structural variations, meaning that they can be applied in numerous extraction processes [36,37]. Moreover, studies have shown that NADES have great selectivity in the extraction of target compounds [36,38,39]. Their noncorrosiveness, low flammability, and low preparation costs make them suitable and convenient for use in industry [33,36]. Anthocyanins are conventionally extracted with mixtures of water and ethanol, methanol, and acetone with associated flammability and toxicity risks [24,39]. The past decade has seen many successful anthocyanin-extraction processes that make use of NADES as the solvents [32,37,40,41]. Dai et al. [39] confirmed that the anthocyanin-extraction efficiency of NADES is similar to that of traditional solvents, and that, moreover, the extracts demonstrate enhanced metabolite stability. To the best of our knowledge, research on using eutectic solvents for the extraction of anthocyanins from blueberry is scarce. For instance, da Silva et al. [42] tested seven different NADES mixtures in the extraction of blueberry fruit anthocyanins in a US bath. Choline chloride:glycerol:citric acid NADES was the optimal eutectic solvent as it gave a depletion yield of 76% compared to an exhaustive conventional protocol. In addition, the chosen eutectic solvent revealed enhanced selectivity for arabinoside anthocyanins.

In this work, we design a novel green extraction methodology for the recovery of high-added-value phytochemicals from blueberry-processing waste. Several NADES mixtures have been tested for anthocyanin extraction and the most sustainable was selected according to its process efficiency, cost, viscosity, and toxicity. The shelf life of extracts prepared in different NADES was estimated. Moreover, percentage polymeric colour was determined to study anthocyanin stability in NADES. UAE and MAE were tested as means with which to enhance anthocyanin recovery, and process kinetics were described using Peleg’s model. The results achieved were compared with those of the conventional extraction methodology. The antiproliferative activity and cytotoxicity of the extracts were then evaluated in in vitro tests on human tumour and skin cell lines. Moreover, the mechanism of cell-growth inhibition and cell death were evaluated using flow cytometry. NADES were recycled and the extracted anthocyanins were recovered using a macroporous resin.

## 2. Materials and Methods

### 2.1. Biomass, Chemicals and Cell Lines

Frozen blueberry (*Vaccinium myrtillus* L.) peels were provided by Indena S.p.a. (Settala, Milano, Italy) as industrial residues. The matrix was stored at −18 °C.

The choline chloride, malic acid, citric acid, lactic acid, glycerol, and glucose used for NADES preparation were ACS grade ≥90%. Ethanol (ACS grade, ≥99%) and hydrochloric acid (37%) were used for anthocyanin extraction, purification, and NADES recycling. Sodium bisulfite (ACS grade, ≥99%) was used to determine total anthocyanin content, while potassium metabisulfite (ACS grade, ≥99%) was used for percentage polymeric colour evaluation. All the above-mentioned chemicals were purchased from Sigma-Aldrich (St. Louis, MO, USA).

The two adherent human cell lines used (HeLa and HaCaT) were purchased from ATCC (Manassas, VA, USA). The Dulbecco’s modified Eagle medium (DMEM) and foetal bovine serum (FBS) used for cell cultivation were purchased from Capricorn (Ebsdorfergrund, Germany). Antibiotic/antimitotic solutions and trypsin-EDTA solutions (0.25%) were purchased from Sigma-Aldrich (St. Louis, MO, USA). The CellTiter 96^®^ AQ_ueous_ One Solution Cell Proliferation assay was purchased from Promega (Madison, WI, USA). The Muse™ Annexin V & Dead Cell Kit was purchased from Merck KGaA (Darmstadt, Germany).

### 2.2. Water Content Determination in Plant Material

The water content in frozen BP was determined by freeze-drying. The plant material was freeze-dried for 24 h using a LyoQuest–85 lyophiliser (Telstar, Madrid, Spain). The analyses were performed in triplicate and results expressed as the average ± SD.

### 2.3. Cryomilling

BP were frozen with liquid nitrogen and subsequently milled in a professional blender (HGBTWTS360, Waring Blender, Stamford CT, USA). The milled BP were stored at −18 °C.

### 2.4. NADES Preparation

NADES were synthesised at fixed molar ratios of choline chloride (ChCl) to hydrogen bond donor (HBD). The two components were placed in appropriate ratios in a round-bottomed glass flask with 22% (*v*/*v*) deionised water. The compounds were stirred and heated to 50 °C for 2 h until a homogeneous transparent colourless liquid was formed. NADES abbreviations and corresponding molar ratios are given in Table 1.

### 2.5. NADES Screening

All the NADES with water contents of 22% were tested for anthocyanin extraction from cryomilled BP to find the optimal HBD. The BP-to-NADES ratio (S/L ratio, hereafter expressed as the mass-to-volume ratio, *w*/*v*) was 1:15 and the final water content in NADES was 25% (*v*/*v*).

The process parameters from a previous investigation into the use of NADES in anthocyanin extraction [36,39] were adapted. In particular, extraction was performed with conventional heating and stirring for 2 h at 55 °C and at 200 rpm. The obtained extract was filtered. Every extraction was performed in triplicate and stored at −18 °C before total anthocyanin content (TAC) determination (see Section 2.7).

### 2.6. Conventional Anthocyanin Extraction

Conventional BP anthocyanin extraction was performed according to a protocol optimised by Dai et al. [39], with some modifications. An acidified hydroalcoholic solution (60% *v*/*v* hydroalcoholic solution with 0.8% *v*/*v* of hydrochloric acid) was mixed with nonmilled BP (S/L 1:5) for 2 h. The extraction temperature was 55 °C and the stirring was set at 200 rpm. The obtained extract was filtered under vacuum. The extraction was performed in triplicate and stored at −18 °C before TAC determination (see Section 2.7).

### 2.7. Total Anthocyanin Content (TAC) Determination

The TAC in the extracts and recovered anthocyanin fraction was determined according to the method described in [45]. A 100 μL volume of extract solution was placed in the test tubes. Samples were sequentially diluted with 60% *v*/*v* ethanol solution containing 0.1% *v*/*v* of hydrochloric acid and 2 mL of 2% *v/v* hydrochloric acid solution. In one parallel test, 400 μL of distilled water was added to the sample solution, and, in the other, 400 μL of 15% *w*/*v* sodium bisulfite solution was added. The resulting solution was mixed thoroughly. After 15 min, the absorbance of the resulting solutions was measured at 520 nm, in a 1 cm quartz cuvette, on a Cary 60 UV-Vis spectrophotometer (Agilent Technologies, Santa Clara, CA, USA), against a blank. The TAC was calculated using the following equation:

(1) $$TAC=875\times \left(D_{1}-D_{2}\right)$$

where D_1_ is the absorbance of the control sample (diluted with distilled water) and D_2_ is the absorbance of the bisulphite bleached sample.

TAC is expressed as anthocyanin mass over the mass of dry BP (*mg/g*). All analyses were performed in triplicate and results expressed as the average ± SD.

### 2.8. Shelf Life of BP Extracts Prepared with Different NADES

The shelf life of the BP extracts was evaluated in order to identify the optimal NADES. The products of the NADES screening were stored between +4 and +8 °C and the TAC was measured in triplicate after 1 week, 3 weeks, and 9 months. Results are expressed as the average ± SD.

### 2.9. Percentage Polymeric Colour (PPC) Determination

Percentage polymeric colour (PPC) determination was used to evaluate the monomeric/polymeric ratios of and anthocyanin-like compounds. The acidified ethanol ChCl:LA, ChCl:MA, and ChCl:Gly extracts that were obtained via the conventional extraction method were evaluated using the method described in [46]. A 2.8 mL volume of the diluted sample was placed in the test tubes, and 200 μL 20% *w/v* of potassium metabisulfite solution was added to one parallel set, and 200 μL of water was added to the other. After 15 min, the absorbance of the resulting solutions was measured at 420 nm, λ_vis-max_, and 700 nm in a 1 cm quartz cuvette, using a Cary 60 UV-Vis spectrophotometer, against a blank. The colour density (CD) of the control sample (treated with water) was calculated as follows:

(2) $$CD=[\left(A_{420\;\mathrm{nm}}-A_{700\;\mathrm{nm}}\right)+\left(A_{\;\lambda \;\mathrm{vis}-\max}-A_{700\;\mathrm{nm}}\right)\times DF$$

where DF is the dilution factor. The polymeric colour (PC) of the bisulphite bleached sample is defined as follows:

(3) $$PC=[\left(A_{420\;\mathrm{nm}}-A_{700\;\mathrm{nm}}\right)+\left(A_{\;\lambda \;\mathrm{vis}-\max}-A_{700\;\mathrm{nm}}\right)\times DF$$

Finally, PPC is calculated according to the following equation:

(4) $$PPC=\frac{PC}{CD}\times 100$$

All analyses were performed in triplicate and results expressed as the average.

### 2.10. MAE Kinetics

MAE was performed in a SynthWAVE (Milestone, Bergamo, Italy), which is a pressure-resistant multimode microwave (MW) reactor that is capable of feeding inert gas (N_2_) into the system. For each test, appropriate purging with nitrogen was carried out three times in order to reduce oxidising degradation. The reaction chamber was finally pressurised with 2 bars of N_2_. All screenings were carried out at 500 W of irradiation, with a heating step of 3 min.

MAE was firstly optimised in terms of the extraction temperature and nonmilled-BP-to-optimal-NADES ratio. Tests at 40, 60, 80, and 100 °C were performed at BP-to-NADES ratios of both 1:20 and 1:30, and the extraction time was kept at 15 min. The crude solutions were filtered and TAC was determined.

After the temperature and BP-to-NADES ratio screenings, MAE kinetics were evaluated under the optimal conditions. The instrumental set-up (cool-down required before the reactor could be opened) meant that ongoing sampling could not be performed. Dedicated extractions were therefore carried out at each sampling time, starting with 2 and 5 min and then progressive increases of 5 min up to 40 min. Crude solutions were filtered under vacuum. Every extraction was performed in triplicate and stored at −18 °C before TAC determination (see Section 2.7). The obtained results, expressed as the average ± SD, were used to describe MAE kinetics using Peleg’s model (Section 2.12).

#### 2.10.1. Conventional Comparison—MAE Efficiency Evaluation

In order to be able to evaluate the role of MW irradiation, the MAE that was optimised as described in Section 2.10 was reproduced using conventional heating and stirring extraction, with both the optimal NADES and acidified ethanol. In detail, the extraction was performed for 15 min at 60 °C and the nonmilled-BP-to-solvent ratio was 1:30. The extracts were then filtered under vacuum. Every extraction was performed in triplicate and stored at −18 °C before TAC determination (see Section 2.7). The obtained results were expressed as the average.

#### 2.10.2. MW Degradation Test

The stability of the anthocyanins was monitored during MW irradiation because of their high sensitivity [47,48]. Firstly, conventional heating and stirring BP extraction was performed according to Section 2.6, using the optimal NADES as the solvent. The extracts were then filtered and the TAC was determined. The obtained extract was then divided into three samples and subjected to MW irradiation. The test lasted for 2 h and the temperature was set at 60 °C. The TAC was then evaluated again expressing the results as the average ± SD.

### 2.11. Ultrasound-Assisted Extraction (UAE) Kinetics

UAE was performed using two different probe systems, which were two immersion horns. The first device (Danacamerini sas, Turin, Italy) was set at 100 W (20 kHz), while the second (HNG-20500-SP, Hainertec, Suzhou, China) supplied 500 W (20 kHz). In both the extraction processes, the nonmilled-BP-to-optimal-NADES ratio was 1:20, the average temperature was around 40 °C, and the sampling of roughly 1 mL was performed every 5 min. Total extraction time was 40 min. Every sample was filtered under vacuum. Every extraction was performed in triplicate and stored at –18 °C before TAC determination (see Section 2.7). The obtained results, expressed as the average ± SD, were used to describe the UAE’s kinetics using Peleg’s model (Section 2.12).

#### 2.11.1. Conventional Comparison—UAE Efficiency Evaluation

To evaluate the role of US irradiation, comparison tests were performed under silent conditions. The UAE that was optimised as described in Section 2.11 was reproduced using conventional heating and stirring extraction, with both optimal NADES and acidified ethanol. In detail, the extraction was performed for 30 min at 40 °C with a nonmilled-BP-to-solvent ratio of 1:20. Both extracts were then filtered under vacuum. Every extraction was performed in triplicate and stored at –18 °C before TAC determination (see Section 2.7). The obtained results were expressed as the average.

#### 2.11.2. US Degradation Test

The stability of anthocyanins under sonication was monitored because of the risk of degradation [47,48]. Firstly, conventional heating and stirring BP extraction was performed, according to Section 2.6, using optimal NADES as the solvent. The obtained extract was filtered and the TAC was evaluated. The same extract was divided into three samples and then submitted to a US degradation test using the harshest conditions available, namely 500 W immersion-horn irradiation. The temperature was maintained at around 40 °C, mimicking that of UAE. Sampling of roughly 1 mL was performed every 4 min. The total test time was 40 min. The TAC was then evaluated again for every sample, expressing the results as the average ± SD.

### 2.12. Peleg’s Model

As mentioned above, UAE and MAE kinetics were described using Peleg’s model [49], which is defined by the following equation:

(5) $$TAC\left(t\right)=\frac{t}{K_{1}+K_{2}\times t}$$

where t is extraction time, K_1_ is Peleg’s rate constant, and K_2_ is Peleg’s capacity constant. Both the kinetic constants were extrapolated from experimental data via the linearisation of Equation (5) (R^2^ ≥ 0.99). From K_1_, the value of the extraction rate at t = t_0_ (B_0_) can be obtained:

(6) $$B_{0}=\frac{1}{K_{1}}$$

Using K_2_, the maximum TAC yield, when t→∞ (Ct→∞), can be calculated:

(7) $$C_{t\to \infty }=\frac{1}{K_{2}}$$

The optimal US power for UAE and the optimal extraction time for UAE and MAE can be determined using the B_0_ and Ct→∞ values.

### 2.13. Anthocyanin Isolation from BP Extracts and NADES Recycling

Anthocyanin isolation from the BP ChCl:LA extract was performed over Sepabeads SP 825L macroporous resins (Mitsubishi Chemical Corporation, Resindion SRL, Milan, Italy). Prior to the separation process, the extract was diluted so that there was more than 50% *v*/*v* water in the NADES. After the resin was prepared using ethanol and water, the extract was loaded. Fraction 1, which contained the recycled NADES, was eluted with 3 bead volumes (BV) of deionised water. The anthocyanins (Fraction 2) were desorbed using 3 BV of 75% *v*/*v* ethanol with 0.1% *v*/*v* of hydrochloric acid. Water from Fraction 1 and ethanol/water from Fraction 2 were then removed in a rotary vacuum evaporator, and freeze-drying was then performed. Finally, recycled NADES and dry anthocyanins were obtained.

Anthocyanin recovery efficiency was evaluated by measuring the TAC in the obtained anthocyanin fraction. Moreover, NADES recycling efficiency was estimated by weighting the obtained recycled NADES.

### 2.14. Antiproliferative Activity and Cytotoxicity Determination

The antiproliferative activity and cytotoxicity of the BP extracts that were prepared in ChCl:LA, both using the above-described enabling technologies and the conventional method, were evaluated in vitro using the CellTiter 96^®^ AQ_ueous_ One Solution Cell Proliferation (MTS) assay, as described in [40]. Two human adherent cell lines were used for this test: cancer HeLa cells derived from the cervical adenocarcinoma and normal human keratinocyte cells (HaCaT). Both cell lines were cultivated in DMEM supplemented with 5% (*v*/*v*) FBS and 1% (*v*/*v*) antibiotic/antimitotic solution and kept in BioLite Petri dishes (Thermo Fisher Scientific, USA) in an incubator with a humidified atmosphere and 5% *v*/*v* CO_2_ at 37 °C. Single tests on the antiproliferative activity and cytotoxicity of the extracts were performed in 96-well plates (Thermo Fisher Scientific, Waltham, MA, USA) that had been seeded with exponentially growing cells at an initial concentration of 3 × 10^4^ cells per well in 100 μL of culture media. After 24 h of incubation, under the cell cultivation conditions, the cells were treated with the extracts. The raw BP extracts that were prepared with ChCl:LA under US and MW and the conventional BP extract were all diluted in the culture medium and then applied to the cells, resulting in final volume ratios of 0.5%, 2% and 5% (*v*/*v*). A double control set was performed: (i) the vehicle-treated cells for ChCl:LA and acidic ethanol in the same volume ratio as the extracts; (ii) nontreated cells. Aiming to verify that the biological activity was not affected by low solvent pH, a dedicated screening was carried out on tumour cells adjusting the extract pH, testing the higher volume ratio previously applied (5% *v*/*v*). Treatment lasted for 72 h in the incubator, followed by the MTS assay. The assay was performed according to the manufacturer’s instructions with a few modifications. A 10 μL volume of MTS reagent was added to each well and the cells were incubated for 3 h; the absorbance was measured at 492 nm on the microplate reader (Tecan, Männedorf, Switzerland). Cell-viability percentage was expressed as the ratio between the absorbances of the treated versus nontreated control cells. The tests were performed in triplicate with four parallels for each volume ratio.

### 2.15. Cell-Death Evaluation by Flow Cytometric Analysis

The quantitative analysis of live, apoptotic, and dead cells that had been treated with the BP extracts—those obtained in ChCl:LA under US and MW and the conventional extract—was carried out on a Muse^®^ Cell Analyser (EMD Millipore Corporation, Burlington, MA, USA) using the Muse™ Annexin V & Dead Cell Kit according to the manufacturer’s specifications. HeLa and HaCaT cells were seeded individually into 6-well culture plates at an initial concentration of 5 × 10^4^ cells mL^−1^ (2 mL per well). After incubation overnight, the cells were treated with BP extracts that had been prepared with ChCl:LA under US and MW and the conventional BP extract, individually, for 72 h. HeLa cells were treated with 0.5 and 5% *v*/*v* of all the aforementioned extracts. The treatment of HaCaT cells was performed with 0.5 and 5% *v*/*v* of US and MW extracts individually and 0.5% *v*/*v* of the conventional extract. After treatment, both the floating and adherent cells were collected using a trypsin-EDTA solution to dissociate the cells from the culture plates and give single-cell suspensions. The collected suspensions were centrifuged (30,000× *g*) for 15 min and the separated cells were suspended in the cell culture medium to adjust the cell concentration according to the manufacturer’s protocol. A 100 μL volume of aliquots of the cell suspension was then added to 100 μL of Muse™ Annexin V & Dead Cell Reagent and incubated in the dark at room temperature (RT) for 20 min. Subsequently, the cells were analysed using the Muse^®^ Cell Analyser. The Muse™ Annexin V & Dead Cell Assay detects phosphatidylserine on the external membranes of apoptotic cells via Annexin V-PE binding, while 7-aminoactinomycin D (7-AAD) is used as a dead-cell marker. Therefore, this assay is able to detect four distinctive cell populations: live (Annexin-V-negative and 7-AAD-negative), early apoptotic (Annexin-V-positive and 7-AAD-negative), late-stage apoptotic (Annexin-V-positive and 7-AAD-positive), and dead cells (Annexin-V-negative and 7-AAD-positive). Each sample was tested in duplicate and each experiment was performed twice. Hence, for every test, four measurements have been collected, ensuring biological reliability.

### 2.16. Statistical Analysis

Statistical analyses were performed using the software Statistica (Statsoft Inc., Tulsa, OK, USA), version 10. Where required, the measurements were processed using Tukey’s HSD test and statistical difference (*p* < 0.05) were designated by lower-case letters.

## 3. Results

### 3.1. Water Content in Plant Material

The average water content in the frozen BP was 56.70 ± 0.19%, *w*/*w*. This value was used to calculate the effective water addition necessary to prepare NADES with 25% *v*/*v* H_2_O content, as reported in Section 2.5.

### 3.2. NADES Screening

**Figure 1:**
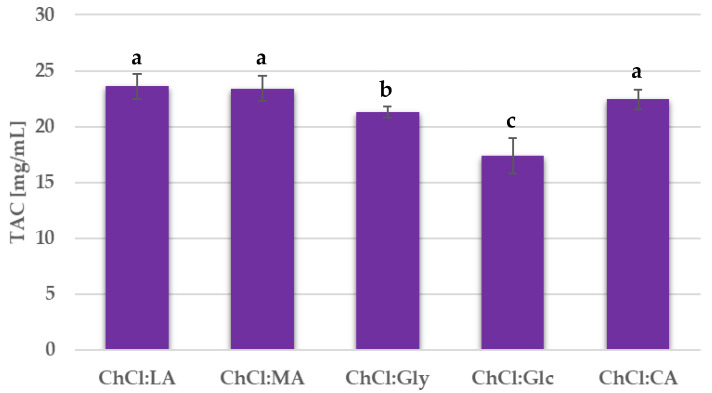
Total anthocyanin content (TAC) for the extracts obtained from NADES screening. Results are expressed as average values ± SD. The presented values followed by different lower-case letters (a–c) are significantly different from each other (*p* < 0.05), as determined by Tukey’s HSD test.

Firstly, different NADES mixtures were tested for BP anthocyanin extraction. The tests were performed with the conventional heating and stirring protocol. In order to avoid the possibility that variations in the viscosities of the different NADES may influence anthocyanin yield, the extraction time was extended to 2 h, and, in addition, the plant material was cryomilled. The temperature was kept at 55 °C during the extraction. It has been reported in the literature that anthocyanin degradation should not occur at this temperature over long extractions [50,51,52]. The TAC for every extraction performed is reported in Figure 1.

### 3.3. Shelf-Life Evaluation of BP Extracts Prepared with Different NADES

The shelf life of the BP extracts prepared using different NADES mixtures was monitored in order to evaluate their stability.

**Figure 2:**
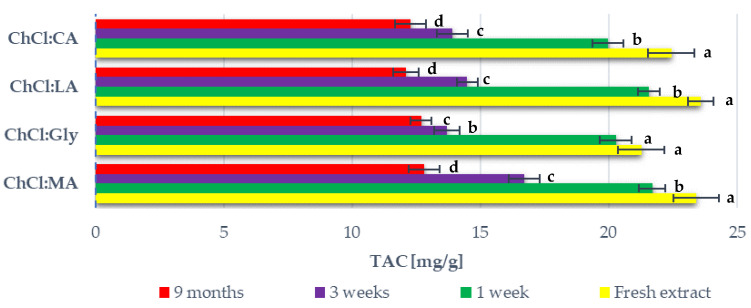
TAC content of blueberry peel (BP) extracts prepared with different NADES mixtures and stored at +4 to +8 °C over 9 months. Results are expressed as average values ± SD. The presented values followed by different lower-case letters (a–d) are significantly different from each other (*p* < 0.05) according to the NADES systems, determined by Tukey’s HSD test.

The extracts that were prepared during the NADES screening (defined as “fresh extract” in Figure 2) were stored at +4 to +8 °C. After one week, three weeks, and nine months, the TAC in the stored extracts was evaluated. The results are reported in Figure 2. Data for the ChCl:Glc extract are not shown as the sample separated after one week.

### 3.4. Conventional Anthocyanin Extraction

Conventional BP anthocyanin extraction was performed in a stirring and heating system using acidified ethanol as the solvent. In order to simplify the overall process, the cryomilling pretreatment was removed as it was irrelevant to the final yields. The same approach was maintained for the following screenings. This extraction gave a TAC of 22.70 mg/g, and this value was set as the benchmark for all further extractions.

### 3.5. Percentage Polymeric Colour (PPC) Determination

Anthocyanin degradation is usually associated with the formation of polymers, and it is therefore possible to evaluate alterations in these metabolites in different solvents using PPC determination. All the extracts were prepared in conventional heating and stirring equipment under the same conditions (2 h, 55 °C, 200 rpm). After filtration, PPC was evaluated. The Ch/Glc system was not taken into account because of its degradation issues, as reported in Section 3.3. The results are shown in Table 2.

### 3.6. Microwave-Assisted Extraction (MAE)

#### 3.6.1. Extraction Kinetics

**Figure 3:**
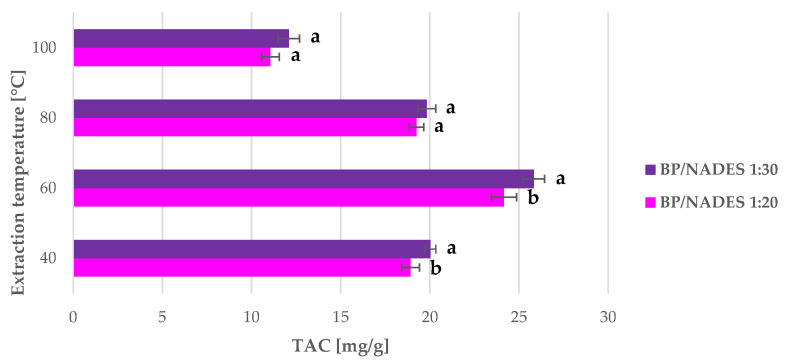
TAC yields of BP anthocyanin microwave-assisted extraction (MAE) under different extraction conditions (S/L ratio and temperature) for 15 min. Results are expressed as average values ± SD. The presented values followed by different lower-case letters (a,b) are significantly different from each other (*p* < 0.05) according to extraction temperature, as determined by Tukey’s HSD test.

All the MAE of nonmilled BP were carried out in a multimode MW reactor under an inert atmosphere (N_2_) to avoid anthocyanin oxidation. Two BP-to-ChCl:LA ratios, namely 1:20 and 1:30, were tested at four different temperatures, 40, 60, 80, and 100 °C. The different solvent ratios were evaluated in consideration of the moderate mass transfer provided by the system due to vials and magnetic stirrer geometries. Lower BP-to-NADES ratios were not considered because of the inefficient matrix stirring. This set of extractions was performed for 15 min, and attention was focused on the other parameters. Results, in terms of TAC, are reported in Figure 3.

**Figure 4:**
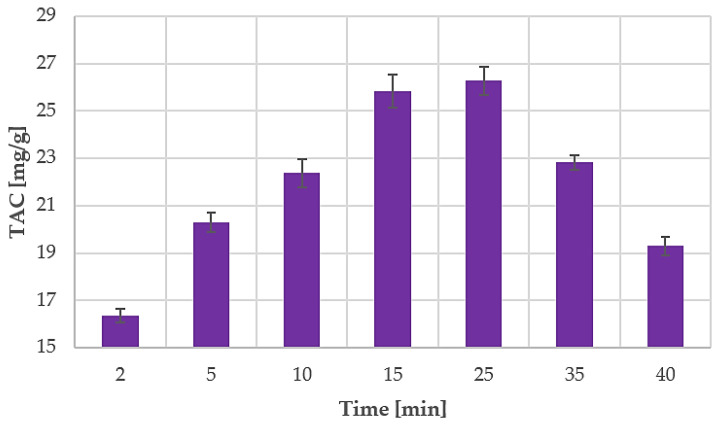
TAC during MAE time, screening with optimised parameters (1 to 30 S/L ratio, 60 °C). Results are expressed as average values ± SD.

The obtained data were used to evaluate anthocyanin MAE kinetics for the best-performing results (60 °C, S/L ratio of 1:30). Sampling during the extraction was not feasible due to the reactor security set-up. The irradiation chamber cannot be opened without prior RT cool-down. A dedicated extraction was therefore carried out for every sample. Optimal MAE parameters were monitored over a time range of 40 min, starting at 2 min and 5 min with subsequent 5 min/step increases. The TAC values for all the performed MAEs are shown in Figure 4.

#### 3.6.2. Conventional Comparison—MAE Efficiency Evaluation

The MAE parameters that led to the highest TAC values were transposed to a heating and stirring system with the aim of evaluating the effect of MW irradiation on BP anthocyanin extraction and comparing it to the conventional extraction methodology. The conventional extractions were performed at 60 °C for 15 min and at an S/L ratio of 1:30, in both ChCl:LA and acidified ethanol, to clarify the solvent effect. The results are reported in Table 3.

#### 3.6.3. MW Degradation Test

**Figure 5:**
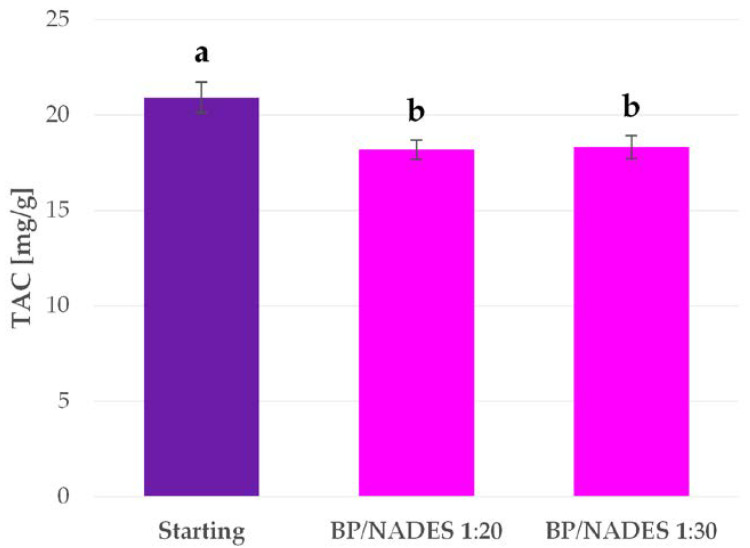
TAC in BP extracts before and after MW irradiation. Results are expressed as average values ± SD. The presented values followed by different lower-case letters (a,b) are significantly different from each other (*p* < 0.05), as determined by Tukey’s HSD test.

TAC stability was monitored over extended irradiation because of the observed degradation that occurred during MAE screening (see Figure 4). A starting, anthocyanin-rich fraction was collected from the conventional protocol with ChCl:LA. After the extraction and subsequent filtration, the extracts were divided into two samples to replicate ratios of 1:20 and 1:30. The tests were carried out with MW irradiation for 2 h at 60 °C under an inert N_2_ atmosphere to avoid oxidative degradation. The initial and final TAC values were determined, and the results are reported in Figure 5.

### 3.7. Ultrasound-Assisted Extraction (UAE)

#### 3.7.1. Extraction Kinetics

The UAE of nonmilled BP anthocyanins was performed using two different US probe systems that supplied 100 W and 500 W, respectively. ChCl:LA, which was previously identified as the optimised solvent, was exploited at a BP-to-NADES ratio of 1:20. This S/L amount was introduced as it was the minimum threshold to allow the transmission of acoustic waves through the medium because of the global system viscosity. Considering the mass-transfer enhancement generated by acoustic cavitation, no other solution consistencies were screened.

**Figure 6:**
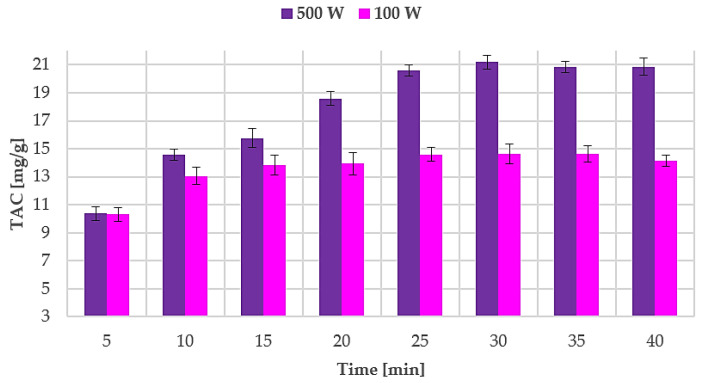
TAC during ultrasound-assisted extraction (UAE) at different powers (100 W and 500 W). Results are expressed as average values ± SD.

Sampling was performed every 5 min and the TAC were determined in order to describe the UAE kinetics and evaluate the optimal extraction time. Results are reported in Figure 6.

#### 3.7.2. Conventional Comparison—UAE Efficiency Evaluation

The optimal UAE conditions were transposed (extraction temperature and time) to a conventional heating and stirring protocol to evaluate the effect of US irradiation. Silent extractions were performed using both ChCl:LA and acidified ethanol in order to clarify the role of the solvent system. The results, in terms of TAC, are reported in Table 4.

#### 3.7.3. US Degradation Test

The incidence of degradation phenomena during extraction was then investigated in the ChCl:LA system under maximum US intensity. For this purpose, a fresh extract was prepared using the conventional protocol (2 h using ChCl:LA). The extract was filtered and instantly subjected to US irradiation and monitored over the 40 min course, which served as the kinetic screening. During the US degradation test, the temperature was maintained below 40 °C in an effort to reproduce extraction conditions. Sampling was performed every 4 min and the TAC was evaluated. The reference TAC of the fresh extract was 20.91 ± 0.52 mg/g, and no degradation was observed during the screening.

### 3.8. Anthocyanin Isolation from BP Extract and NADES Recycling

The low vapour pressure of NADES means that three methods for solvent reuse and target compound recovery are feasible: liquid–liquid extraction using another solvent, adsorption chromatography, and the addition of an antisolvent [36,53].

In this study, ChCl:LA was recycled by adsorption chromatography, as described in [36]. The extract was diluted in the chromatography to give more than 50% of water to the NADES in order to enhance the separation of the anthocyanins and the NADES solvents; the hydrogen bonds between NADES constituents are broken when the water content is above 50% *v*/*v* [54]. The availability of the anthocyanins for adsorption was enhanced, and there was a dramatic decrease in viscosity. The diluted extract was loaded onto the microporous resin, which has a high affinity towards polyphenols, and the anthocyanins adsorbed onto this resin, while the NADES simply eluted with water. Subsequently, the water fraction was removed by freeze-drying, which allowed ChCl:LA to be recovered. Finally, 79.48% of the NADES was recovered and was sufficiently pure for recycling in further extractions. After being separated from the eutectic solvent, the anthocyanins were desorbed from the resin using acidified ethanol (0.1% *v*/*v* hydrochloric acid); 72.55% of the TAC was recovered in this process.

### 3.9. Extract Antiproliferative Activity and Cytotoxicity Evaluation

**Figure 7:**
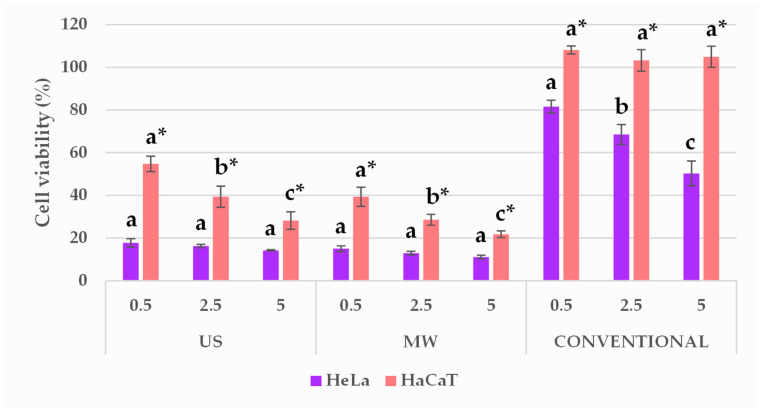
Effect of BP extracts in the final volume ratios of 0.5, 2.5, and 5% (*v*/*v*), prepared using the different methodologies, on HeLa and HaCaT cell viability. Results are expressed as average values ± SD. Statistically different data (*p* < 0.05) are designated by lower-case letters (a–c or a *–c *, HeLa and HaCaT, respectively), according to volume ratios, whereas the statistical difference among the cell lines are marked by a graphical signature (*).

The optimal BP extracts that were prepared using MAE and UAE and the conventional ethanolic extracts were all tested for their antiproliferative activity in the tumour cell line (HeLa) in comparison with a normal human keratinocyte cell line (HaCaT) by MTS assays. The obtained results are shown in Figure 7. The vehicle-treated control showed that cell viability was not significantly affected by the addition of solvents in 0.5% to 5% (*v*/*v*); thus, those results are not depicted in Figure 7. Therefore, the cell viability (%) was expressed as a percentage of treated cells vs. nontreated control, and statistical difference was denoted among extracts and among cell lines used.

### 3.10. Cell-Death Evaluation

**Figure 8:**
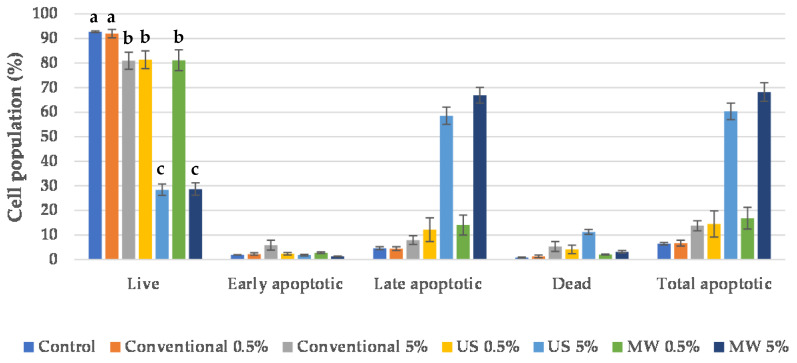
Cell-death evaluation for HeLa cells after treatment with BP extracts. Results are expressed as average values ± SD. Statistically different data according to cell population type among control and obtained extracts (*p* < 0.05) are designated by lower-case letters (a–d).

**Figure 9:**
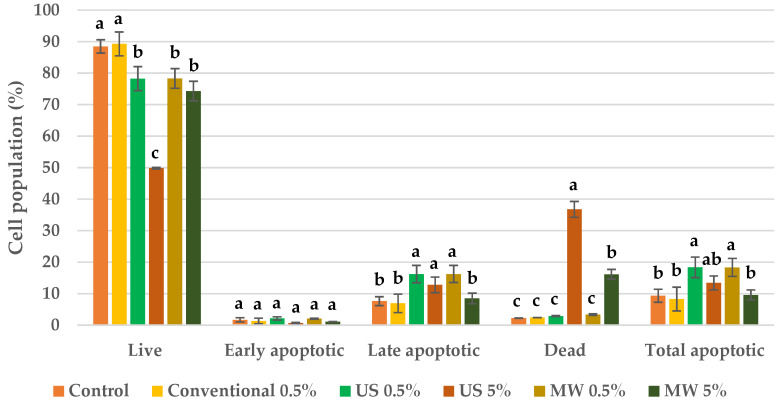
Cell-death evaluation for HaCaT cells after treatment with BP extracts. Results are expressed as average values ± SD. Statistically different data according to cell population type among control and obtained extracts (*p* < 0.05) are designated by lower-case letters (a–c).

Whereas the antiproliferative evaluation demonstrated the activity of the BP extracts, the cell-death type was evaluated on a Muse^®^ Cell Analyser by flow cytometry using a Muse™ Annexin V & Dead Cell Kit. The results are depicted in Figure 8; Figure 9 for both HeLa and HaCaT cell lines, respectively.

## 4. Discussion

### 4.1. NADES Screening

Preliminary NADES screening was conducted on five different choline-based systems under conventional extraction conditions (see Figure 1). ChCl:MA and ChCl:LA gave the highest, but very similar, TAC values of 23.41 and 23.59 mg/g, respectively. The optimal NADES was selected on the basis of viscosity, price, and cytotoxicity. The HBD prices were reviewed on Sigma-Aldrich (St. Louis, MI, USA). A 1 kg total of LA costs €55.14 and the price of the same mass of MA is €49.00. These NADES, therefore, have comparable preparation costs.

Although the viscosity of all of the NADES mixtures can be decreased by the addition of water, ChCl:LA appears to be generally less viscous, and is, hence, more easily incorporated into industrial processes.

Radošević et al. [55] evaluated the chemical cytotoxicity of numerous NADES and cholinium-based ionic liquids on channel catfish ovary cells. The study proved that ChCl:MA has a large influence on cell viability, while ChCl:LA showed barely any effect at all. Zhao et al. [56] investigated the toxicity of different NADES on several bacteria species (*Escherichia coli, Staphylococcus aureus, Salmonella enteritidis, Listeria monocytogenes*). ChCl:MA was shown to be one of the most toxic NADES of those tested. The increasing interest in the sustainable production of lactic acid and the development of suitable biorefinery approaches was an aspect that was evaluated further [57]. Based on the data found in literature, ChCl:LA was therefore selected as the optimal solvent system and was adopted for the subsequent investigations.

### 4.2. Shelf-Life Evaluation of BP Extracts Prepared with Different NADES

The shelf life of the BP extracts that were obtained from the preliminary NADES screening was investigated at one week, three weeks, and nine months (see Figure 2). The stability, in terms of TAC, was very similar for all the monitored systems. A significant TAC loss was noted between the first and third weeks of storage. In between the third week and nine months, there was no significant TAC loss. Similar behaviour has been observed by Srivastava et al. [21]. The stability of the anthocyanins and other polyphenols in the blueberry extract that was prepared using water was monitored over 60 days. Significant anthocyanin degradation occurred in the extracts stored at 6 °C from Day 15 to Day 30. Afterwards, there was no significant degradation.

The type of NADES did not have a significant effect on anthocyanin stability. After nine months of storage, the TAC for all the extracts was more or less half that of the initial TAC. In particular, the average TAC loss for the monitored extracts was 44.92 ± 3.45%. Laleh et al. [58] prepared blueberry anthocyanin extracts using an acidified ethanol solution and they were stored at 5 °C for 84 days (i.e., 12 weeks). Anthocyanin stability varied greatly with blueberry species. The TAC loss varied from 10.22 to even 57.81%. Moldovan et al. [59] studied anthocyanin stability in cranberry bush fruit extracts that were prepared in water and ethanol and stored at 2 °C. The solvent acidity effect was also monitored. The highest anthocyanin half-life was observed in water that was acidified at pH 3 and was 48.12 days. The next highest anthocyanin stability was found in ethanol that was acidified at pH 3. However, the TAC was halved to 22.21 days. Conversely, Tao et al. [60] noted a different trend. They performed the UAE of wine lees using an ethanol/water solution and stored the extract for 30 days at 4 °C. No degradation was observed. However, this result may not be representative in terms of the solvent’s effect on anthocyanin stability as the ethanol was evaporated from the extract before storage. Panić et al. [36] monitored anthocyanin stability in grape pomace extracts at 4 °C for 60 days. The extracts were prepared using acidified ethanol, ChCl:CA and ChCl:proline:MA. ChCl:CA showed the highest stabilising capacity, with 14% degradation being observed after 60 days of storage, whereas this value was 70% *v*/*v* for acidified ethanol and ChCl:proline:MA. The literature therefore indicates that the extraction solvent has a significant effect on anthocyanin stability, although information on anthocyanin-extract stability over long term storage is currently quite scarce. The results obtained herein on the shelf life of BP extracts offer a deeper view of anthocyanin stability in different NADES media.

### 4.3. Percentage Polymeric Colour (PPC) Determination

When degraded, monomeric anthocyanins are susceptible to polymer formation [61]. Hence, high PC values can be used as an indicator of anthocyanin degradation. In fact, PPC was determined to evaluate the monomeric/polymeric ratios of anthocyanins and anthocyanin-like compounds in extracts prepared using the different solvents. The effect of the solvent on metabolite alteration was therefore evaluated. The lowest PPC was detected for acidified ethanol. ChCl:CA, ChCl:LA, and ChCl:MA gave very similar PPC values, which were quite comparable to those of the acidified ethanol extract. The ChCl:Gly extract gave a dramatically higher PPC value. These results can most likely be attributed to the solvent pH. Based on their constituents, it is clear that lactic-, malic-, and citric-based NADES have quite an acidic pH, which is favourable for anthocyanin extraction and preservation, whereas glycerol moves toward a neutral or basic pH, and is therefore unsuitable for these purposes. ChCl:CA, ChCl:LA, and ChCl:MA can easily replace traditional acidified ethanol.

The demand for biologically derived anthocyanins is constantly growing. Moreover, industrial processes entail a number of considerations for not only process efficiency but also cost, robustness, safety, and environmental impact. Hence, one of the aims of our work was to evaluate two nonconventional extraction technologies for anthocyanin extraction from the residues of the blueberry production chain. In fact, UAE and MAE were investigated with the aim of enhancing extraction yields and reducing extraction time. Biocompatible and bio-derived NADES were applied, defined after screening (see Section 4.1) for both extraction techniques. These methodologies were compared with the conventional ones.

### 4.4. Microwave-Assisted Extraction (MAE)

#### 4.4.1. Extraction Kinetics

Preliminary tests were performed by investigating the role that temperature and S/L play in BP MAE (see Figure 3). In all cases, the extractions performed with a matrix-to-NADES ratio of 1:30 gave the best TAC yields. Regarding temperature, the maximum TAC was achieved at 60 °C, and a decrease was visible at higher temperatures. The optimal MAE parameters were therefore a BP-to-NADES ratio of 1:30 and temperature of 60 °C. Under these conditions, a TAC yield of 25.83 mg/g was achieved, which is more than 12% higher than that of the benchmark protocol (55 °C, 2 h, acidified ethanol as solvent). The significantly higher extraction yields and extremely low extraction times can be attributed to the process-intensification effect of MW. In addition, their chemical characteristics mean that they are well suited to MAE [36]. Extraction efficiencies can be enhanced by this combination of green solvent and MW.

Extraction at 40 °C at a ratio of 1:30 yielded 20.03 mg/g TAC, which is quite near to the benchmark yield. The low yields of the MAE performed at 80 and 100 °C suggest the occurrence of anthocyanin degradation at these temperatures. Zheng et al. [62] investigated anthocyanin MAE from blueberry powder, using acidified ethanol as the solvent. In this study, metabolite degradation was observed at temperatures of approximately above 50 °C, and was influenced by ethanol concentration and process time.

The optimal temperature and S/L ratio, which were defined in the initial screening (see Figure 3), were used to determine extraction kinetics. Periodic sampling was performed up to 40 min, and the TAC values are reported in Figure 4. The yield after only 5 min was extremely high at 20.30 mg/g, which is quite close to the result obtained in the benchmark extraction (22.70 mg/g, see Section 3.4). After 25 min of extraction, the anthocyanin concentration greatly decreased, which suggests that the degradation phenomena occurred. As already mentioned, anthocyanins are degradable phytochemicals whose stability is strongly dependent on pH, temperature, light, oxygen exposure, and the presence of some enzymes [16,17]. Metabolite stability during MAE does not only depend on the extraction temperature but also involves the energy irradiated towards the sample. Some studies have reported that faster anthocyanin degradation occurs under MW treatment at 700 W than in a conventional thermal bath at 98 °C. [63,64] All tests were performed in a pH 3.5 buffer solution. Their hypothesis was that the MW irradiation induces Baeyer–Villiger oxidation via the nucleophilic attack of hydrogen peroxide, which can cause rapid anthocyanin degradation. However, the degradation mechanism in our case requires further investigation as NADES was used as the solvent. 

Peleg’s model was used to describe MAE kinetics. As Peleg’s hyperbolic equation does not take degradation phenomena into consideration, only extraction times up to 25 min were considered for linearisation. The obtained model was defined by the following equation:

(8) $$TAC\left(t\right)=\frac{t}{0.0652+0.0355\times t}$$

B_0_ at t = t_0_ had a value of 15.34 mg/g min. C_t→__∞_ was 28.17 mg/g.

**Figure 10:**
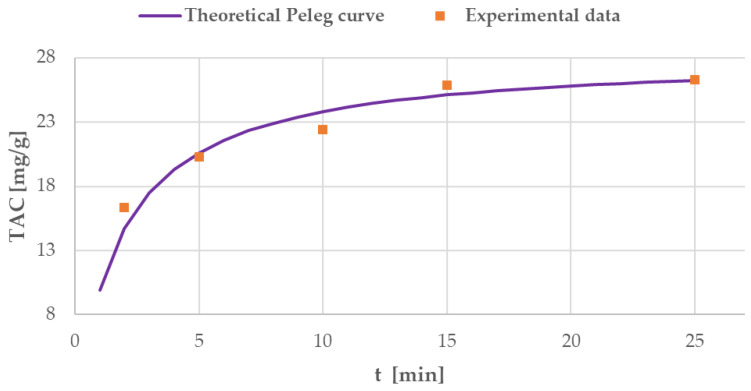
Curve obtained from MAE experimental data and elaborated using Peleg’s model.

The obtained theoretical curve and the experimental data for MAE are shown in Figure 10.

The extraction rate B_0_ was found to be particularly high, leading to 20.30 mg/g TAC after only 5 min of extraction. The maximum TAC yield in _t→__∞_, C_t→__∞_ had a significantly higher value than that of the benchmark extraction. Process time optimisation for BP MAE was performed using the theoretical curve, approaching C_t→__∞_, and the optimised time was found to be 15 min. Afterwards, the TAC did not increase notably, proving that degradation phenomena had a limited impact. The experimental TAC yield at 15 min was 25.83 mg/g, whilst the theoretical yield was 25.03 mg/g. Hence, in summary, the optimal MAE parameters are a BP-to-ChCl:LA ratio of 1:30, a temperature of 60 °C, and 15 min of extraction.

#### 4.4.2. Conventional Comparison—MAE Efficiency Evaluation

The best-performing parameters for MAE were then reproduced in a conventional system (see Table 3), thus making it possible to isolate the contribution of the solvent, temperature, and S/L ratio, and evaluate the technique’s efficiency. Optimal MAE gave a much higher TAC yield than the conventional extractions that were carried out using NADES and even acidified ethanol. These results strongly confirm the process-intensification capabilities of MW. The combination of MW and NADES offers drastic yield increases and cuts down on process time.

#### 4.4.3. MW Degradation Test

The MAE trend observed for BP at 60 °C suggests that anthocyanin degradation occurs. The phenomenon was therefore investigated by subjecting a known concentration of metabolites to MW irradiation. For this purpose, a starting sample was collected from the conventional protocol: this process provided an extract whose components had never been subjected to irradiation. It was therefore possible to avoid underestimating the degradation phenomena, including those of any highly-sensitive compounds. A freshly prepared extract was used to exclude the influence of any possible matrix enzymes or any other matrix compounds that could enhance or reduce anthocyanin stability.

Both S/L ratios were tested, and anthocyanin decreases of 13.06% and 12.38% were observed for the 1-to-20 and 1-to-30 ratios, respectively. It can be stated that a higher quantity of ChCl:LA did not have a significant effect on compound stability. When the MW anthocyanin extraction kinetics were monitored between 25 and 40 min, 26.56% of the anthocyanins were degraded. This result suggests that the presence of a matrix may have an effect on anthocyanin stability. For example, Yousefi et al. [65] investigated anthocyanin degradation in pomegranate juice concentrate. This study confirmed faster colour and anthocyanin alteration when the juice was highly concentrated. In addition, it is well known that enzyme activity and sugar content have an influence on the stability of these metabolites [61].

### 4.5. Ultrasound-Assisted Extraction (UAE)

#### 4.5.1. Extraction Kinetics

The results reported for UAE power screening at different times (see Figure 6) have shown stable extraction trends that are apparently not affected by degradation phenomena. The obtained yields were then processed, according to Peleg’s model, to extrapolate kinetics dependencies. For the probe operating at 100 W, the model was as follows:

(9) $$TAC\left(t\right)=\frac{t}{0.1089+0.0659\times t}$$

B_0_ at t = t_0_ had a value of 9.18 mg/g min. C_t→__∞_ was 15.17 mg/g.

For UAE at 500 W, the obtained model was defined by the following equation:

(10) $$TAC\left(t\right)=\frac{t}{0.2318+0.0414\times t}$$

B_0_ at t = t_0_ and C_t→__∞_ were 4.31 mg/g min and 24.15 mg/g, respectively.

**Figure 11:**
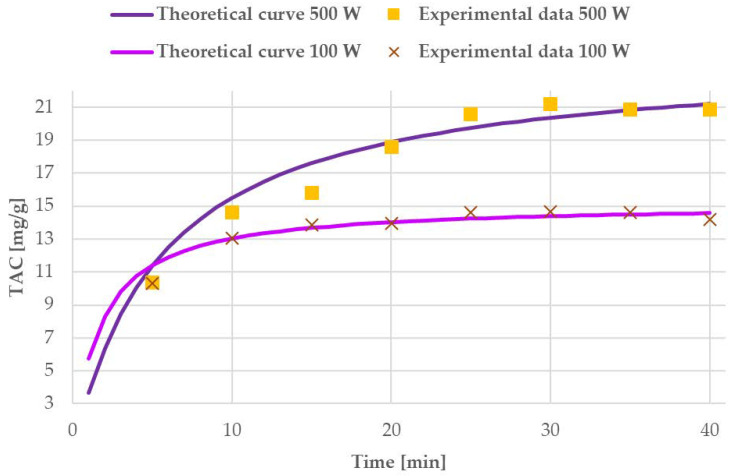
Experimental data and curves resulting from Peleg’s model for 100 W and 500 W US systems.

The experimental data and theoretical modelled curves are presented in Figure 11, for both US systems.

From the theoretical and experimental curves in Figure 11, it is possible to see that the US probe working at 500 W gives a much higher TAC from the first 10 min of the process onwards. The average extraction rate, labelled B_0_, is twice that of the system operating at 100 W (9.18 mg/g min vs. 4.31 mg/g min). These values suggest that the weaker US probe gives a faster increase in TAC yield, but that, on the other hand, it rapidly achieves a lower extraction steady state. In other terms, 500 W intensity led to halved TAC increase over time, but this is counterbalanced by the highest maximum yield. In particular, the parameter that defines maximum TAC (C_t→__∞_, when t→∞) confirmed that the maximum TAC for the milder UAE is one-third lower than that of the harsher conditions (15.17 mg/g, vs. 24.15 mg/g). The extraction trend is clearly described by the different shapes of the initial section of the curves (first 10 min) in Figure 11. Even though the extraction rate was faster for the 100 W system, the probe operating at 500 W was selected as giving the best performance due to the final maximum TAC yield in a moderate span of time. 

Hence, the optimal process time for the UAE at 500 W was determined from the theoretical curve of Peleg’s model. In particular, the closest TAC value to C_t→__∞_ was achieved at 30 min. At this time, the experimental yield was 21.18 mg/g, which can be compared to the theoretical value of 20.36 mg/g. After this time, no significant TAC increase was detected. Therefore, the optimal UAE was fixed to 30 min of irradiation at 500 W (~40 °C).

The optimised protocol gave a very similar result to that of the benchmark extraction, which was performed for 2 h using acidified ethanol. However, the extraction time of UAE was significantly shorter and the process was carried out using a much greener solvent.

#### 4.5.2. Conventional Comparison—UAE Efficiency Evaluation

As performed for MAE, the best-performing UAE parameters were reproduced in a conventional system, and the contributions of solvent, temperature, and S/L ratio were isolated, allowing the technique’s efficiency to be evaluated. Silent extraction performed with ChCl:LA gave an approximately halved TAC yield compared to that of the UAE reference. The effect of US is strongly pronounced and can be explained by enhancements in mass transfer. The high viscosity of NADES strongly affects extraction efficiency, and cavitation was demonstrated to be a suitable technology to overcome the issue.

Concerning the evaluation of the solvent system, it was possible to observe how silent extraction with acidified ethanol gave a yield that was quite similar to that of optimal UAE. The explored bio-derived ChCl:LA therefore appears to be competitive with the conventional solvent, without any strong inorganic acid being involved. Moreover, UAE usually provides lower energy consumption than extractions that make use of conventional conductive heating.

Furthermore, the removal of a chlorine-based strong inorganic acid appears to be crucial. This environmentally friendly extraction protocol is also a cost-effective approach, as dedicated equipment is required to handle and apply hydrochloric acid. Ethanol, besides being a taxed solvent, requires explosive atmospheres (ATEX) and antiflame plants.

#### 4.5.3. US Degradation Test

The stability issues that were observed during MAE suggest that anthocyanin degradation may even occur during UAE due to the extreme physical conditions generated by the collapse of cavitation bubbles [66]. Thus, a dedicated investigation was performed at the highest sonication power available (500 W). As in the MW degradation test, the starting sample was obtained from the conventional protocol to avoid US-dependent deterioration during the extraction phase. Nevertheless, no significant TAC alterations were observed throughout the duration of the screening. Chen et al. [67] reported that anthocyanin degradation occurred during raspberry UAE when high power was applied, even though the extraction was performed with acidified ethanol, which should act as a stabiliser. The unchanged TAC in our case, despite the high US irradiation being applied, may suggest that ChCl:LA has a stabilisation effect on the BP extract at the working temperature. This hypothesis is supported by Dai et al. [39], who confirmed the stabilisation activity of eutectic solvents toward anthocyanins. For example, the half-life time of cyanidin (used as a reference) was more than three times higher in NADES lactic acid/glucose than in acidified ethanol, at 60 °C.

### 4.6. Anthocyanin Isolation from BP Extract and NADES Recycling

As mentioned above, reuse and recycling are some of the fundamental concepts of the circular economy approach. NADES can be easily recycled thanks to their advantageous physicochemical properties. Furthermore, the isolation of an anthocyanin-rich fraction may pave the way for a wide range of possible nutraceutical and pharmaceutical applications. In this work, a preliminary study of a resin was performed, and a NADES recycling of 79.48% was achieved with a final TAC recovery of 72.55%. The data in the literature also report the loss of some metabolites during the recovery process. Wang et al. [11] performed polyphenol recovery from fig leaf extracts obtained in Gly:xylose:fructose NADES using a microporous resin. The recovery of individual polyphenolic compounds varied from 75.3 to 85.5%. Zhuang et al. [68] used different microporous resins for the recovery of flavonoids from *Platycladi cacumen* ChCl:LA extracts. The recorded recovery efficiency varied between 77.44% and 98.92% depending on the flavonoid monitored and the resin used.

In our work, the TAC loss during recovery can be explained by the partial elution of anthocyanins together with the NADES/water fraction, as the recycled NADES had a slightly pink colour. However, the presence of anthocyanins in the recycled ChCl:LA should not be a problem, as this solvent should be recycled in the BP extraction process. Dai et al. [43] reported the coelution of polyphenols with NADES ChCl:sucrose in the absorption chromatography process. Panić et al. [36] reported colour retention in recycled ChCl:CA NADES after anthocyanin recovery from grape pomace extract. ChCl:LA traces were visible in the dry anthocyanin fraction after freeze-drying. Similarly, Dai et al. [43] performed the recovery of extractables obtained using LA:Glu NADES, and reported that LA was retained on the microporous resin together with the polyphenols. As LA has shown low cytotoxicity [55,69], its presence in purified extracts should not be an issue when it comes to implementation in food and cosmetic formulations. This exploratory result encourages further investigation into the use of purification steps with resins.

### 4.7. Extract Antiproliferative Activity and Cytotoxicity Evaluation

Blueberries are one of the richest possible sources of anthocyanins [70], whose chemoprotective properties have been extensively reviewed in in vitro studies and animal models [15]. However, data on the biological activity of polyphenols, including their antiproliferative activity, in NADES are still quite scarce. Generally speaking, DES composed of ChCl and organic acid HBDs display low toxicity [71]. Moreover, both ChCl and LA are substances that are generally recognised as safe (GRAS) by the Food and Drug Administration (FDA) [72]. It therefore seems that this NADES could easily be implemented in nutraceutical and pharmaceutical formulations.

In our present work, an MTS assay was performed on HeLa and HaCaT cells to investigate the antiproliferative activity of the optimised BP extracts, in accordance with their extraction technologies. The conventional ethanolic sample was taken as a reference. The results reported in Figure 7 confirmed that NADES can emphasise the biological activity of the extracts. In comparison with the conventional BP extract, the US and MW extracts possess significantly stronger antiproliferative activities. Moreover, the conventional extract added to the culture medium had a considerably higher TAC concentration (mg anthocyanins/L) with respect to the ChCl:LA extracts, as reported in Table 5, underlining the reported positive effect of NADES on the investigated biological activity.

However, the conventional extract is not cytotoxic for human skin cells (HaCaT), while the US and MW extract demonstrate cytotoxicity in HaCaT cells. Nevertheless, the effects of the US and MW extracts on tumour-cell growth are considerably greater than their effect on keratinocytes. Different activity trends have been seen in different cell lines:

- In HeLa cells, the US and MW extracts showed almost unvaried inhibitory effects regardless of the applied volume ratio, while the conventional sample showed dose-dependent behaviour.
- The HaCaT cell line was characterised by less pronounced growth inhibition than in HeLa cells, which is dose-dependent. The hydroalcoholic extract did not affect cell growth.

Comparable results were obtained by Radošević et al. [73], who evaluated the antiproliferative activity of grape pomace extracts that were prepared using different NADES and MeOH. The conventional MeOH extract demonstrated very low antiproliferative activity, unlike the sample prepared with ChCl:MA. Similar results were achieved in tumour cells by Panić et al. [40], who investigated grape and olive pomace extracts, obtained by means of NADES systems. Both substrates were prepared in ChCl:CA, using EtOH as the benchmark. The latter showed much lower inhibitory effects than the eutectic/extract mixtures.

**Figure 12:**
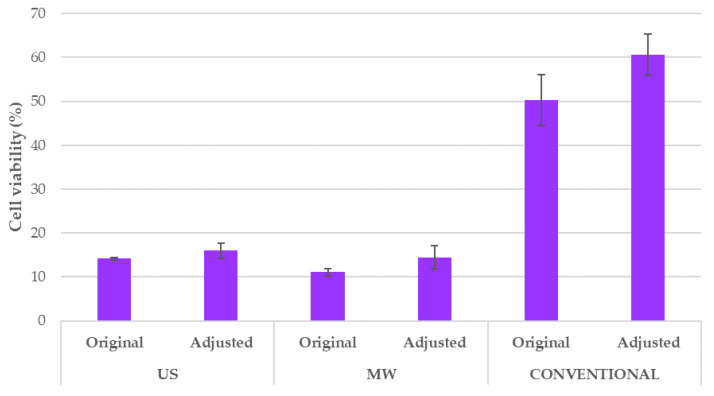
HeLa cell viability after treatment with original extracts vs. pH-adjusted extracts for different extraction techniques. Results are expressed as average values ± SD. There is no statistical difference between the results, as determined by Tukey’s HSD test.

Some further considerations regarding the acidic pH of the tested BP samples are required. When the extract is added, the culture medium is not able to maintain physiological pH, even at the lowest volume ratios. Aiming to verify that the observed activity arose from the extract composition independently by the pH, a dedicated test was performed. Hence, the pH of the obtained acidic extracts was adjusted up to pH 6 and the viability of HeLa cells was determined. Cell treatment with adjusted pH gave comparable results to the original (Figure 12). Small differences in cell viability were observed and not statistically significant.

In support of these results, several studies have shown that an in vivo extracellular matrix of the tumour-cell microenvironment has a more acidic pH than normal tissue [74,75]. For normal human cells, p53-dependent apoptosis pathways are activated in environments with pH lower than 7, and this finally results in cell death. Tumour cells, on the other hand, have mutant p53 genes, and usually exhibit a maximum proliferation rate in a relatively acidic medium, at around pH 6.8 [74]. Since the results presented on Figure 12 showed that extracts with adjusted pH also have comparable antiproliferative effect on the tumour cell line, it can be excluded that the acidic pH of the original extracts caused the observed inhibition of the cell growth.

### 4.8. Cell-Death Evaluation

Mammalian cell-death types are most widely defined into two major classes: Apoptosis and necrosis [76]. Necrosis is an unregulated (accidental) type of cell death and is caused by acute physicochemical injuries. Apoptosis is the most commonly studied programmed cell death. It maintains the equilibrium between growing and dead cells and prevents damaged and malignant cell growth. Nevertheless, other programmed cell-death types, autophagy and programmed necrosis, are known [77,78,79,80]. Besides uncontrolled cell proliferation, one of the prominent characteristics of cancer cells is their resistance to apoptosis, making it one of the most essential factors for the monitoring of chemoprotective effects [13].

Once the antiproliferative activity mentioned in Section 4.7 was verified, flow cytometry was used to discover whether BP extracts act via a predominant cell-death mechanism. This approach was applied to both HeLa and HaCaT cell lines (see Figure 8; Figure 9, respectively). For the HeLa cell line, the conventional EtOH extract, added at a 0.5% *v*/*v*, did not have a significant effect on cell population, while the higher concentration (5% *v*/*v*) caused a slight increase in apoptosis. For the US and MW extracts prepared in ChCl:LA, low concentrations (0.5% *v*/*v*) led to a negligible increase in apoptotic cells. Nevertheless, the apoptotic cell population was significantly increased, up to 60%, when these extracts were added at a 5% *v*/*v*. Therefore, it can be concluded that apoptosis plays the main role in tumour-cell death when BP extracts are added.

Similarly to tumour cells, low-ratio extract additions did not have an effect on the HaCaT cell population, regardless of the extract source (conventional, ChCl:LA in US and MW, see Figure 9). However, cell death by accidental necrosis occurred when the US and MW extracts were added to the culture medium at a 5% *v*/*v*. Specifically, US prompted a great increase in the necrotic cell population, whereas a slight increase was observed for the MW sample.

Such a difference in the type of cell death between tumour and normal cells can be explained by the already mentioned fact that tumour cells are less sensitive to acidic pH of its surrounding media, whereas for normal HaCaT cells acidification of culture media due to addition of 5% volume US and MW is probably a physicochemical injury, which led to necrosis.

The cell-death evaluation results confirmed that the conventional BP extract does not possess antiproliferative activity that is as strong as that of the BP extracts prepared in NADES. Moreover, the primary cause of tumour-cell death is apoptosis. Unfortunately, the BP extracts prepared using enabling technologies prompted an increase in necrotic cells in normal skin cells, which was much more prominent in the extracts prepared using US. However, the apoptotic cell population in the HeLa cell line was much higher than the necrotic cell population in normal skin cells, especially with the MW extract, implying that these extracts could serve as innovative agents in cancer prevention and treatment. Certainly, to confirm this potential application, in vivo tests are required to further evaluate the chemoprotective and anticancer effects of the investigated systems.

## 5. Conclusions

Bioactive compounds from plant materials are currently gaining increasing amounts of attention, mainly because of their health-promoting benefits. At the same time, since environmental pollution has become a global problem, the exploitation of wastes and byproducts has become a favourable alternative to their disposal. Hence, various plant residues can be exploited as sources of phytochemicals. In this study, the residues of blueberry processing have therefore been used as a source of anthocyanins, which are valuable metabolites that possess a wide range of biological activity.

Academia and industry are both challenged to develop new green extraction methodologies that can reduce environmental impact. The present study addressed this topic with two strategies:

- Investigating the use of NADES, a novel class of sustainable solvents;
- Evaluating the process intensification of two enabling technologies, specifically MW and US.

Specifically, five NADES have been tested in conventional BP anthocyanin extraction, with an acidified hydroalcoholic solution as the benchmark. The shelf life of the extracts was monitored over nine months at between +4 and +8 °C, which confirmed that all the samples have similar stability, except the glucose-based one. Moreover, a supplementary evaluation of solvent-dependent degradation was performed by PPC. This test showed that ChCl:LA, ChCl:MA, and ChCl:CA provide similar anthocyanin stability compared to the hydroalcoholic reference, which highlights them as promising candidates to replace conventional solvents. ChCl:LA was finally selected as the most suitable NADES based on extraction efficiency, cost, viscosity, and toxicity.

In order to pursue higher process efficiency, two nonconventional technologies, namely MAE and UAE, have been explored for use with ChCl:LA. The extraction kinetics was described using Peleg’s model and the optimal parameters were determined. MAE led to 25.83 mg/g TAC in 15 min at 60 °C, with a BP-to-ChCl:LA ratio of 1:30. UAE achieved 21.18 mg/g TAC after 30 min of sonication at 500 W power. The role played by the technologies and NADES was verified via comparisons with conventional procedures. The results supported the innovative approaches and showed that they were able to enhance productivity and save time. A preliminary study on the MW- and US-mediated degradation of anthocyanins was included, and a matrix effect, which is predominant in MAE, was observed.

In order to fulfil the requirements of the circular economy approach, an explorative test on anthocyanin concentration and NADES recycling was performed using a microporous resin. A final TAC recovery of 72.55% was achieved, together with solvent reuse of 79.48%. These results strengthen the case for extending the use of resins for purification purposes.

Finally, the antiproliferative activity of conventional and ChCl:LA (MAE and UAE) extracts was determined in vitro in the human tumour cell line. The HeLa cell line was tested and compared to human skin cells (HaCaT) in MTS assays, and it was found that the MAE and UAE samples possess significantly stronger antiproliferative activity than the conventional BP extract. Moreover, the growth inhibition effect is considerably greater in tumour cells than in skin cells. These results confirm that NADES are able to emphasise the biological effects of recovered phytochemicals. Cell-death type was determined by flow cytometry, using a Muse™ Annexin V & Dead Cell Kit, to provide insight into the cell-growth inhibition mechanism. The experiment detected that apoptosis was the primary tumour-cell death cause indicating the possibility to induce destruction of tumour cells by obtained extracts. Nevertheless, further in vivo tests are required to better understand and verify the proposed biological activity.

In conclusion, this study has developed two new extraction methods for the recovery of anthocyanin-rich extracts with enhanced antiproliferative activity. Moreover, the results demonstrated how US and MW can provide extensive extraction efficiency intensification compared to the conventional extraction protocol. These results pave the way for the development of new pharmacologically active compounds that are prepared using innovative green procedures.

## Figures and Tables

**Table 1:** Natural deep eutectic solvents (NADES) used in this study and their abbreviations and molar ratios.

NADES	Abbreviation	Molar Ratio	Reference
Choline chloride:malic acid	ChCl:MA	1.5:1	[32,40]
Choline chloride:citric acid	ChCl:CA	2:1	[33,36]
Choline chloride:lactic acid	ChCl:LA	1:1	[33,43]
Choline chloride:glycerol	ChCl:Gly	1:2	[32]
Choline chloride:glucose	ChCl:Glc	1:1	[39,44]

**Table 2:** Percentage polymeric colour (PPC) in extracts prepared using different solvents.

Extraction Solvent	PPC (%)
Acidified EtOH	16.37
ChCl:CA	19.02
ChCl:LA	19.90
ChCl:MA	19.10
ChCl:Gly	65.71

**Table 3:** TAC in the ChCl:LA and acidified ethanol conventional extracts and the optimal microwave (MW) extract.

Extract	TAC (mg/g)
Optimal MAE	25.83
ChCl:LA, conventional	15.88
Acidified EtOH, conventional	19.42

**Table 4:** TAC in ChCl:LA and acidified ethanol silent extracts and optimal ultrasound (US) extract.

Extract	TAC (mg/g)
ChCl:LA UAE (optimised)	21.18
ChCl:LA, silent	13.86
Acidified EtOH, silent	20.92

**Table 5:** TAC added to the culture medium for HeLa and HaCaT treatment. Reported for every volumetric ratio tested (0.5, 2.5, and 5 % *v*/*v*).

Extract	TAC in 0.5% (*v*/*v*) (mg/L)	TAC in 2.5% (*v*/*v*) (mg/L)	TAC in 5% (*v*/*v*) (mg/L)
US	5.30	26.48	52.95
MW	4.31	21.53	43.05
Conventional	7.57	37.83	75.67
